# Radiotherapy for Genitourinary Cancer

**DOI:** 10.3390/curroncol32090513

**Published:** 2025-09-15

**Authors:** Natsuo Tomita, Takuya Koie

**Affiliations:** 1 Department of Radiology, Nagoya City University Graduate School of Medical Sciences, 1 Kawasumi, Mizuho-cho, Mizuho-ku, Nagoya 467-8601, Japan; 2 Department of Urology, Gifu University Graduate School of Medicine, Gifu 501-1194, Japan; koie.takuya.h2@f.gifu-u.ac.jp

As the second most common cancer in men, prostate cancer was diagnosed in 1.47 million men worldwide in 2022 alone [1]. Radiotherapy is one of the main treatments for common genitourinary cancers like prostate and bladder cancer [1], and is widely used as an adjuvant therapy with surgery or as a curative treatment in itself. With radiotherapy for genitourinary cancer being the focus of this Special Issue, and prostate cancer being the most prevalent of these malignancies, 10 of the 13 papers published in this herein are related to prostate cancer. The first contribution focuses on radioligand therapy for prostate-specific membrane antigen (PSMA-RLT), providing promising results from the VISION trial, and predicts that this treatment is expected to gain rapid, widespread adoption across the world [2]. Having already attracted the attention of many *Current Oncology* readers, a review by Hoshi et al.^1^ summarizes various findings on the efficacy and issues of PSMA-RLT, where the authors discuss optimal candidates for the therapy. 

The following four contributions are dedicated to external beam radiation therapy (EBRT) for prostate cancer. Numakura et al.^2^ illustrate the effects of dose escalation and high-precision radiotherapy for localized prostate cancer, such as intensity-modulated radiation (IMRT) and image-guided radiation therapy (IGRT), as well as summarizing clinical trials on brachytherapy and particle beam therapy. A notable consideration arising from this work is whether these high-precision radiotherapy can increase the dose in cases of postoperative recurrence [3]. Fujii et al.^3^ investigated the relationship between adverse events and dose parameters in stereotactic body radiation therapy (SBRT) for localized prostate cancer, using data from 145 cases at a single institution. Notably, toxicity after prostate SBRT with 32–36 Gy in four fractions was manageable, and it has recently been demonstrated that five-fraction SBRT is as effective as conventional radiotherapy in terms of biochemical or clinical recurrence; this means it may be a viable treatment option for patients with low- to intermediate-risk localized prostate cancer [4]. Ustumi et al.^4^ analyzed clinical predictors of early biochemical recurrence in high-risk prostate cancer patients who underwent carbon ion radiation therapy and androgen deprivation therapy. Nuijens et al.’s^5^ narrative review examines patient-related factors associated with late toxicity following EBRT for pelvic tumors, with a focus on patients with prostate and cervical cancers.

The following five papers focus on brachytherapy for localized prostate cancer. Papers 6, 7, and 8 are from a Gifu University research group. Ito et al.^6^ investigated clinical, treatment, and dose parameters related to urinary toxicities after low-dose-rate (LDR) brachytherapy performed at a single institution. Additionally, Yamaguchi et al.^7^ examined the usefulness of seed density as a predictor of iodine-125 seed migration in prostate cancer patients. Takeuchi et al.^8^ redefined biochemical recurrence-free survival after LDR brachytherapy, while Guhlich et al.^9^ analyzed dose distributions using a radiobiological model to determine the optimal combination of EBRT and high-dose-rate (HDR) brachytherapy. Finally, Kato et al.^10^ reviewed the findings on LDR brachytherapy, including salvage therapy and the latest brachytherapy technologies.

The remaining three papers focus on bladder cancer, upper urinary tract carcinoma, and primary malignant tumors that arise from the episiotomy site. Houssiau et al.^11^ examined the impact of c-MET expression on treatment outcomes in bladder cancer. Upper urinary tract urothelial carcinoma is a rare tumor that accounts for only 5% of urothelial carcinomas, and the role of adjuvant radiotherapy remains controversial. Zalay et al.^12^ conducted a systematic review and meta-analysis of the existing literature to further explore the potential role of adjuvant radiotherapy for this rare tumor. Palicelli et al.^13^ conducted a systematic literature review according to the PRISMA guidelines on primary malignant tumors arising from episiotomy sites.

Technological innovations in radiotherapy are steadily occurring. The dissemination and adoption of new approaches to radiotherapy in the management of genitourinary malignancies, such as prostate cancer, are essential to improving patient outcomes [5,6,7,8]. Although not featured in this Special Issue, it is encouraging that evidence for hypofractionated radiation therapy has become clearer, improving treatment convenience for patients [5,6]. In addition, it is expected that evidence will be established regarding the optimization of radiotherapy for newly diagnosed metastatic prostate cancer [7] and the significance of dose escalation through techniques such as focal boost [8]. The contributions in this Special Issue are expected to refine the role of radiotherapy in genitourinary cancer and encourage further clinical and translational progress.
